# Metabolic perturbation studies using a Nash Equilibrium model of liver machine perfusion: modeling oxidative stress and effect of glutathione supplementation

**DOI:** 10.3389/fsysb.2023.1260315

**Published:** 2024-01-08

**Authors:** Angelo Lucia, Korkut Uygun

**Affiliations:** ^1^ Department of Chemical Engineering, University of Rhode Island, Kingston, RI, United States; ^2^ Center for Engineering in Medicine and Surgery, Harvard School of Medicine, Shriners Children’s, Massachusetts General Hospital, Boston, MA, United States

**Keywords:** liver metabolism, machine perfusion, oxidative stress, inflammation, glutathione supplementation

## Abstract

The current clinical standard of Static Cold Storage (SCS) which involves preservation on ice (about +4°C) in a hypoxic state limits storage to a few hours for metabolically active tissues such as the liver and the heart. This period of hypoxia during can generate superoxide and other free radicals from purine metabolism, a well-established component of ischemia/reperfusion injury (IRI). Machine perfusion is at the cutting edge of organ preservation, which provides a functional, oxygenated preservation modality that can avoid/attenuate IRI. In clinical application, perfusion usually follows a period of SCS. This presentation of oxygen following hypoxia can lead to superoxide and hydrogen peroxide generation, but machine perfusion also allows manipulation of the temperature profiles and supply of antioxidant treatments, which could be used to minimize such issues. However, metabolomic data is difficult to gather, and there are currently no mathematical models present to allow rational design of experiments or guide clinical practice. In this article, the effects of a gradual warming temperature policy and glutathione supplementation to minimize oxidative stress are studied. An optimal gradual warming temperature policy for mid-thermic machine perfusion of a liver metabolic model is determined using a combination of Nash Equilibrium and Monte Carlo optimization. Using this optimal gradual warming temperature policy, minimum GSH requirements to maintain hydrogen peroxide concentrations in the normal region are calculated using a different Monte Carlo optimization methodology. In addition, the dynamic behavior of key metabolites and cofactors are determined. Results show that the minimum GSH requirement increases and that the ratio of GSH/GSSG decreases with increasing hydrogen peroxide concentration. In addition, at high concentrations of hydrogen peroxide it is shown that cytochrome C undergoes dysfunction leading to a decrease in useful oxygen consumption and ATP synthesis from the electron transport chain and an overall reduction in the energy charge for the liver cells.

## 1 Introduction

The current clinical standard of Static Cold Storage (SCS) which involves preservation on ice (about +4°C) in a hypoxic state limits storage to a few hours for metabolically active tissues such as the liver and the heart. This rather restrictive limit creates major logistical constraints that compound donor organ shortage and presents a major obstacle to on-demand tissue availability and global organ sharing. Given SCS by definition involves creating hypoxia for the organ, the field is pushing *ex situ* Machine Perfusion (MP) as a functional preservation method, providing oxygen and nutrients in the duration the organ is outside a body. While clinical successes are extremely promising and demonstrate MP as superior to SCS, there is a limited understanding about the metabolic processes underlying the improvement. In particular the organ recovery process invariably involves some amount of cold ischemia for the organs prior to initiation of MP, which makes it likely that the grafts experience some level of ischemia/reperfusion injury (IRI). There is very limited data on the metabolic trajectory an organ follows during this critical period, and moreover many metabolic processes occur very quickly, in the order of minutes, which make it problematic if not impossible to capture some changes experimentally. Mathematical models could illuminate these metabolic processes during the organ preservation period which in turn could guide experimental design and clinical practice.

There is extensive established evidence in the open literature that documents IRI (Salaris, et al., 1991; Shibuya et al., 1997; Galaris et al., 2006; Hines et al., 2013; Hines and Grisham, 2011; Tang et al., 2022; Bardallo et al., 2022) and the potential for complications that lead to organ transplantation failure. While IRI is a complex, multistage process, all articles on the subject cite the presence of reactive oxygen species (ROS) such as superoxide ( $\left.O_{2}^{-}\right)$ , and hydroxyl ion ( ${OH}^{-}$ ) free radicals and hydrogen peroxide as the underlying cause of inflammation, mitochondrial dysfunction, and ischemia-reperfusion injury. ROS represent a double-edged sword in that normal levels of the free radicals are necessary for signaling and elimination of harmful microorganisms while excessive amounts can lead to cell proliferation in cancers and apoptosis. The most common types of ROS damage in organ preservation stem from 1) the breakdown of purines during hypoxia (e.g., in static cold storage) and/or 2) excessive superoxide production from electron leakage from the electron transport chain during reperfusion (i.e., the re-introduction of blood flow), resulting in excessive hydrogen peroxide production, glutathione (GSH) depletion, and mitochondrial dysfunction.

During normal respiration, superoxide has a concentration in the range of $10^{-11}$ to $10^{-12}$ molar (M) (Miriyala et al., 2011) and is converted into hydrogen peroxide in the mitochondria by superoxide dismutase (SOD2, EC 1.15.1.1)

$$2O_{2}^{-}+2H^{+}=H_{2}O_{2}+O_{2}$$ (1)

Hydrogen peroxide is then converted into water and oxygen

$${2H}_{2}O_{2}={2H}_{2}O+O_{2}$$ (2)

by the enzyme catalase (EC 1.11.1.6). It is generally agreed that the mitochondrial matrix concentrations of $H_{2}O_{2}$ [also denoted $\left[H_{2}O_{2}\right.$ ]] under physiological conditions is in the range $10^{-9}-10^{-8}$ M. See, Sies (2017) and Valdez et al. (2018). Concentrations of $10^{-7}$ M and higher can lead to cell damage. Because hydrogen peroxide concentrations are more easily measured than free radical concentrations H_2_O_2_ is often used as a biomarker for oxidative stress and inflammation (Sies, 2017). Please note that in this work, we use $H_{2}O_{2}$ as the marker of inflammation, which allows using a metabolic model without the need to include NFkB signaling, immune cell recruitment among other processes, which we consider outside of scope for this manuscript.

Under normal physiological conditions, the antioxidant glutathione (GSH, $C_{10}H_{18}N_{3}O_{6}S^{+1}$ ), which is transported from the cytosol to the mitochondria, is a primary scavenger of hydrogen peroxide. In the presence of glutathione peroxidase (EC 1.11.1.9) GSH and H_2_O_2_ undergo the following reaction

$$2C_{10}H_{18}N_{3}O_{6}S^{+1}+H_{2}O_{2}=C_{20}H_{34}N_{6}O_{12}S_{2}^{+2}+2H_{2}O$$ (3)

and produce water and glutathione disulfide (GSSG). Glutathione depletion and/or the concentration ratio [GSH]/[GSSG] are also often used as an indicator of oxidative stress. However, note that twice as much glutathione is needed to remove 1 mol (mol) of hydrogen peroxide and that 2 mol of water are produced for each mole of H_2_O_2_ removed. This is an important fact that can lead to glutathione depletion when an organ is under oxidative stress or inflammation. This, in turn, can result in elevated levels of $H_{2}O_{2}$ and suggests that exogenous glutathione supplementation could be used to alleviate oxidative stress during reperfusion. Others have suggested the use of antioxidants (Salaris et al., 1991; Hines et al., 2013) to combat oxidative stress.

The objective of this article is to test if the recently developed Nash Equilibrium (NE) modeling approach (Lucia and DiMaggio, 2019) to metabolic network analysis can be used to study oxidative stress and provide quantitative information for antioxidant supplementation in liver machine perfusion. This article is organized as follows. Section 2 presents our current model of liver metabolism. The logistics of the numerical simulations are described in Section 3. In Section 4, perturbations of hydrogen peroxide are used to simulate oxidative stress/inflammation during static cold storage (SCS) and liver machine perfusion. The goals of this work were to use Nash Equilibrium and Monte Carlo optimization to determine 1) the optimal temperature (or gradual warming) policy following SCS and 2) the minimum level of GSH supplementation to keep hydrogen peroxide concentration in the normal range during gradual warming. Select plots of changes in oxygen and glutathione consumption, hydrogen peroxide concentration, ATP synthesis, glutathione disulfide production, and [GSH]/[GSSG] for various levels of oxidative stress and inflammation during MP are presented along with illustrations of the time evolution of key metabolites and a discussion of the numerical simulation results. Conclusions of the study are drawn in Section 5.

## 2 A metabolic model of the liver

**FIGURE 1:**
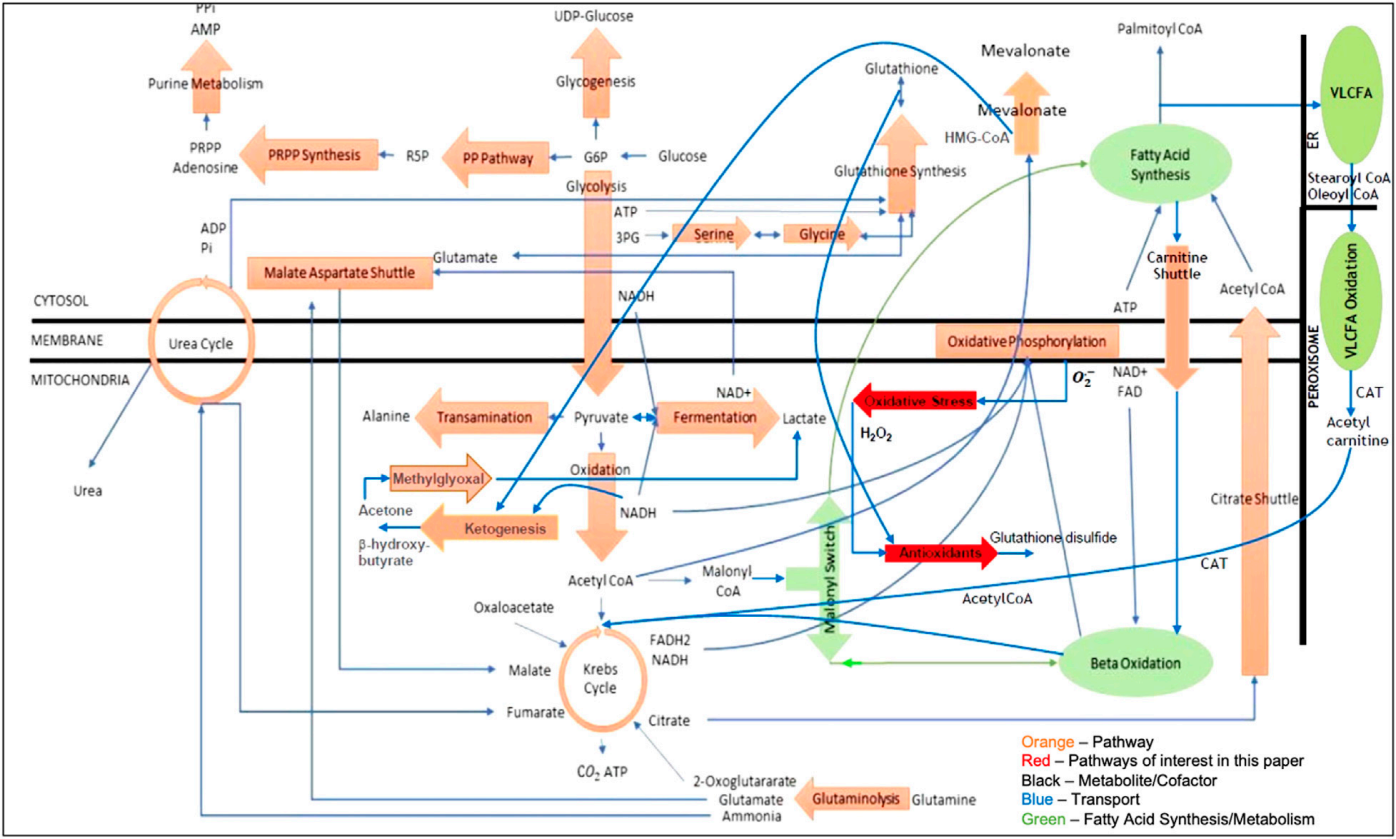
Metabolic network model of the liver.

Figure 1 is the network model of the liver that was used in this article. It contains five cellular compartments, twenty-six pathways, sixty-nine chemical reactions (including Eqs 1–3 shown in the introduction), three hundred thirty-seven metabolites and cofactors, two hundred and twenty-four atom (charge) balances, and eighty-seven standard state Gibbs free energies and enthalpies of formation, which are the only parameters used in the model.

All key model assumptions and simplifications are described in the Supplementary Table S1. The two pathways shown in bright red in Figure 1 represent the major pathways for oxidative stress and antioxidant behavior and are the main focus of this article.

## 3 The basis of numerical simulations using Nash Equilibrium

### 3.1 Nash Equilibrium modeling

The modeling approach used in this manuscript is based on the Nash equilibrium (NE) framework for determining unknown fluxes throughout a given network recently developed by Lucia and coworkers (Lucia and DiMaggio, 2019). The application of NE to liver metabolism is discussed in detail in Lucia et al. (2022) and will be briefly summarized here:

1) Enzymes are players in a multi-player game.
2) Each player (enzyme) minimizes the Gibbs free energy of the reaction it catalyzes subject to element mass balances.
3) The goal of the metabolic network is to find the best overall solution given the natural competition for nutrients among enzymes.

### 3.2 Formulation

The NE formulation for an arbitrary metabolic network is a rigorous, first principles approach that does not ignore or constrain accumulation/depletion of intermediate metabolites and cofactors.

The NE formulation is given by a collection of j=1,2,…,N nonlinear programming (NLP) sub-problems of the form

$$\left[minimize\frac{G_{j}\left(\nu_{j}\right)}{RT}subject\;to\;conservation\;of\;mass,v_{j}^{*}\right]$$ (4)

where $\frac{G_{j}}{RT}$ , the dimensionless Gibbs free energy, is the objective function associated with the appropriate enzyme that catalyzes one or more reactions at a given node j in the network, R is the universal gas constant, and T is absolute temperature. The conservation of mass constraints are elemental mass balances that often involve charged species and, as in other approaches, $\nu_{j}$ represents the fluxes of metabolic material at node j . Finally, the vector, $\nu_{-j}^{*}$ denotes the optimal fluxes of all other sub-problems, k=1,2,…,j−1,j+1,…,N . The Gibbs free energy for sub-problem j is given by

$$\frac{G_{j}}{RT}=\sum_{i=1}^{C_{j}}x_{ij}\left[\frac{{∆G}_{ij}^{0}}{RT}+\ln\left(\mu_{ij}\right)\right]$$ (5)

where $∆G_{ij}^{0}$ are the standard Gibbs free energies of reaction at 25°C for the metabolic reactions associated with sub-problem j , $x_{ij}$ are mole fractions, which are calculated from the fluxes, $\mu_{ij}$ are chemical potentials, i is a component index, and $C_{j}$ is the number of components associated with a sub-problem j in the network.

Temperature effects are included using the Gibbs-Helmholtz equation, which is given by

$$\frac{∆G_{ij}^{0}\left(T\right)}{RT}=\frac{∆G_{ij}^{0}\left(T_{0}\right)}{RT_{0}}+\frac{∆H_{ij}^{0}\left(T_{0}\right)}{R}\left[\frac{\left(T-T_{0}\right)}{TT_{0}}\right]$$ (6)

where $T_{0}$ is the reference temperature (usually 298 K), T is the temperature at which the reaction takes place (say 310 or 337 K), and $∆H_{ij}^{0}\left(T_{0}\right)$ is the standard enthalpy change of reaction i at node j in the network. All standard Gibbs free energy changes due to reaction, $∆G_{ij}^{R0}\left(T_{0}\right)$ , and the enthalpy changes due to reaction, $∆H_{ij}^{R0}\left(T_{0}\right)$ , can be computed from Gibbs free energies and enthalpies of formation and reaction stoichiometry. For example,

$$∆G_{ij}^{R0}=\sum_{k=1}^{n_{p}\left(i,j\right)}S_{k}∆G_{f,ijk}^{0}-\sum_{k=1}^{n_{r}\left(i,j\right)}S_{k}∆G_{f,ijk}^{0}$$ (7)

where the $S_{k}$ ’s are the actual stoichiometric numbers based on conversion and $n_{p}\left(i,j\right)$ and $n_{r}\left(i,j\right)$ are the number of products and number of reactants respectively associated with reaction i and node j .

All necessary Gibbs free energies of formation data has been assembled in a local database and was taken from eQuilibrator (http://equilibrator.weizmann.ac.il/). Feedbacks within any pathway are converged using successive substitution. Also, any number of enzyme-substrate reactions can be explicitly included in the model and require that:

1) The RCSB Protein Data Bank (https://www.rcsb.org/pdb/home/) for accurate enzyme and/or enzyme-substrate structure data.
2) AutoDock or 1-Click-Docking (https://mcule.com) to compute the necessary Gibbs free energies of binding (or binding affinities) for all key enzymes.

### 3.3 Nash Equilibrium pseudo-algorithm

The Nash Equilibrium algorithm is iterative.

1) Define a metabolic network.
2) Identify all pathways (and species: metabolites, cofactors, enzymes), pathway connections, and transport fluxes in the given metabolic network.
3) Read properties for all species (i.e., Gibbs free energies and heats of formation, molecular weights, etc.).
4) Break all feedback and transport fluxes in the network and estimate the initial values of these fluxes.
5) Determine an initial steady-state solution.
6) Perturb the metabolic network to elicit a response.
7) Solve each NLP sub-problem given by Eqs 4–7 for the minimum Gibbs free energy for all unknown fluxes, including feedback and transport fluxes.
8) Compare the new calculated transport fluxes with previously estimated flux values.
  a) If the 2-norm of the difference is within a certain tolerance, $\epsilon =10^{-3}$ , or a preset number of time steps have been taken, stop.
  b) If not, replace estimated feedback and transport fluxes with the calculated ones; go to step 7.

The numerical simulations of the liver model shown in Figure 1 consisted of

1) University of Wisconsin (UW) flush of the liver followed by 6 h of static cold storage at 4°C. Following Southard et al. (1990), the UW flush used in our simulations contains only the important constituents of the solution—adenosine and glutathione. The UW solution approximation used in this work is shown in Table 1. There is superoxide present primarily from purine metabolism to hypoxanthine, xanthine, and uric acid by xanthine oxidase since the cells are in a hypoxic state during SCS. However, note that the UW solution contains the antioxidant glutathione so there should be some glutathione present in the cells following static cold storage.
2) Machine Perfusion at 16 °C (i.e., mid-thermic MP or MMP) for 8 h using Williams Medium E (WME). The approximation of Williams Medium E used in this work is shown in Table 2. During MP, the majority of superoxide synthesis comes from leakage of electrons in the electron transport chain and is estimated at 1−2 % of the oxygen consumed (Miriyala et al., 2011). In this work we assume that there is a 2% leakage of O_2_ from the mitochondria to form superoxide.

**TABLE 1:** UW solution components included in the model.

Compound	Southard et al. (1990) (mM)	Amount (nmol/cell)
Glutathione	3	0.0003
Adenosine	5	0.0005
Water		5.54

**TABLE 2:** Williams medium E solution components included in the model.

Component	Conc (g/L)	(g/50 mL)^a^	MW	Amount (nmol/cell)	Molarity (mM)
Glucose	2	0.1	180.06	1.2340	11.074
Glutamine	0.292	0.0146	146.07	0.2221	1.9990
Glutathione	0.00005	0.0000025	307.08	$2.22\times 10^{-5}$	0.00247
L-serine	0.01	0.005	105.04	0.1057	0.9520
Glycine	0.05	0.0025	75.03	0.0740	0.6664
L-alanine	0.09	0.0045	89.09	0.1122	1.0102
L-arginine	0.05	0.0025	174.11	0.0319	0.2872
L-aspartate	0.03	0.0015	133.04	0.0250	0.2254
L-cysteine	0.04	0.0020	121.02	0.0367	0.3306
Bicarbonate	2	0.1	60.99	3.6432	32.6943

^a^ Actual amounts of WME, constituents used in machine perfusion simulations.

The mathematical model of the network shown in Figure 1 served as the basis for the numerical studies in this paper. More specifically, all reactions were solved to (some desired level of) chemical equilibrium using Gibbs free energy minimization in an inner loop while the transport fluxes shown in blue in Figure 1 were solved in an outer loop. In addition, while osmotic equilibrium is assumed in the model, chemical equilibrium between intra- and extracellular metabolites is not invoked. The same network model and algorithm were used for both SCS and MP. Algorithmic details can be found in Lucia et al. (2022).

## 4 Numerical perturbation studies of oxidative stress and inflammation

The numerical studies in this article included the following sequence of simulations/optimizations:

1) A baseline simulation of liver metabolism that included static cold storage and machine perfusion.
2) Monte Carlo temperature policy optimization of mid-thermic machine perfusion (MMP).
3) Monte Carlo optimization of GHS in the medium for addressing oxidative stress/inflammation.

All results presented in this section represent samples of the many simulations/optimizations conducted for this work, with simulations being repeated >100 times in each case as previously detailed in Lucia et al., 2022. Note that the optimizations of temperature policy and GSH supplementation were decoupled. All computations were performed on a Dell Vostro 5510 laptop using the Lahey-Fujitsu (LF95) compiler.

### 4.1 Baseline simulation

The baseline for all numerical perturbation studies of ischemia-reperfusion injury were as follows:

1) The liver was flushed with UW solution and then simulated for 6 h of SCS.
2) The resulting SCS solution was used as the starting point for the MP simulations/optimizations using Williams Medium E.
3) All MMP simulations/optimizations were run for 8 h.

During all MMP simulations/optimizations, several key biomarkers were used to ensure that the NE solution was physiologically meaningful. In particular, the NE methodology considered a solution to be physiologically meaningful if and only if the following conditions were satisfied:

1) Glycolysis produced a net amount of ATP.
2) The ratio of net ATP from the Krebs cycle to glucose consumed was between 0 and 2.
3) Oxidative phosphorylation synthesized ATP.
4) The malonyl-CoA synthesis reaction only proceeded in the forward direction because it is the committed step in fatty acid synthesis and therefore is irreversible.
5) Synthesis of fatty acids consumed ATP.
6) A net amount of glutathione disulfide was produced.

### 4.2 Monte Carlo temperature policy optimization

An initial MMP temperature policy of 16 °C was selected. Monte Carlo temperature policy (or warming rate) optimization (Eqs 8–10) was conducted following the procedure described in Lucia and Uygun (2022) and briefly summarized here. The objective function used for the constrained temperature policy optimization reflects the viability criteria in Laing et al. (2017), the need for bile production, and a measure of liver energy state and had the form:

$$R=w_{1}\left|Glu\right|+w_{2}ATP+w_{3}Mev+w_{4}EC$$ (8)

where *Glu* is glucose consumption, *ATP* is net ATP production, *Mev* is mevalonate production (a measure of bile production), *EC* is energy charge, and *w*_*1*_—*w*_*4*_ are weights. Following Laing et al. (2017), the liver viability constraints used in this study were:

$$\left[\text{lactate}\right]\leq 2.3\text{ mM and pH}>7.3$$ (9)

The optimization problem was defined as follows:

$$\max_{T\left(t\right)}R:\left[\text{lactate}\right]\leq 2.3\text{ mM},\text{pH}>7.3$$ (10)

where $T\left(t\right)$ is discrete and given by $T\left(t\right)=\left\{T_{1},T_{2},\ldots ,T_{N}\right\}$ and N is the number of discrete time steps. Monte Carlo acceptance criteria required that 1) R increase, 2) $T_{1}\leq T_{2}\leq \ldots \leq T_{N}$ , and 3) all six of the conditions described in Section 4.1 be satisfied. Table 3 shows the concentrations of key metabolites and cofactors along with pH and energy charge for SCS followed by MMP using Williams medium E for both conventional MMP and MMP with temperature policy optimization (i.e., OPT MMP which stands for optimal gradual warming MMP).

**TABLE 3:** Baseline solutions: 6 h of SCS followed by 8 h of MMP and OPT MMP.

Quantity	Value		
	SCS	MMP	OPT MMP
Glutathione (nmol/cell)^a^	0.3	$2.22\times 10^{-5}$	$2.22\times 10^{-5}$
Oxygen consumed (nmol/cell)	0	2.064	22.432
Superoxide (M)	$3.988\times 10^{-14}$	$2.494\times 10^{-11}$	$6.026\times 10^{-12}$
Hydrogen peroxide (M)^b^	$3.017\times 10^{-9}$	$1.468\times 10^{-8}$	$1.485\times 10^{-8}$
Net change in H_2_O_2_ (nmol/cell)^c^	$0.7{\times 10}^{-10}$	$1.288\times 10^{-5}$	$1.304\times 10^{-5}$
Glutathione (mM)	7.602 × 10^−2^	5.956 × 10^−3^	8.981 × 10^−3^
Net change in GHS (nmol/cell)	−0.234	−0.063	−0.068
Net GSSG synthesis (nmol/cell)	$3.123\times 10^{-9}$	$1.496\times 10^{-5}$	$1.481\times 10^{-4}$
ATP (mM)	0.837	4.169	5.360
Net change in ATP (nmol/cell)	−1.246	0.814	2.398
Net change in bile (nmol/cell)	0.037	$-1.030\times 10^{-4}$	$2.543\times 10^{-3}$
pH	7.56	7.71	7.67
Energy charge	0.5071	0.6038	0.6124

^a^ GHS, in SCS, comes from UW, solution.

^b^ MMP, and 8 h of OPT MMP, were perturbed with 2.5 × 10−7 nmol/cell of hydrogen peroxide following SCS, to elicit a response.

^c^ Consumed in SCS, by GSH, in UW, solution.

**FIGURE 2:**
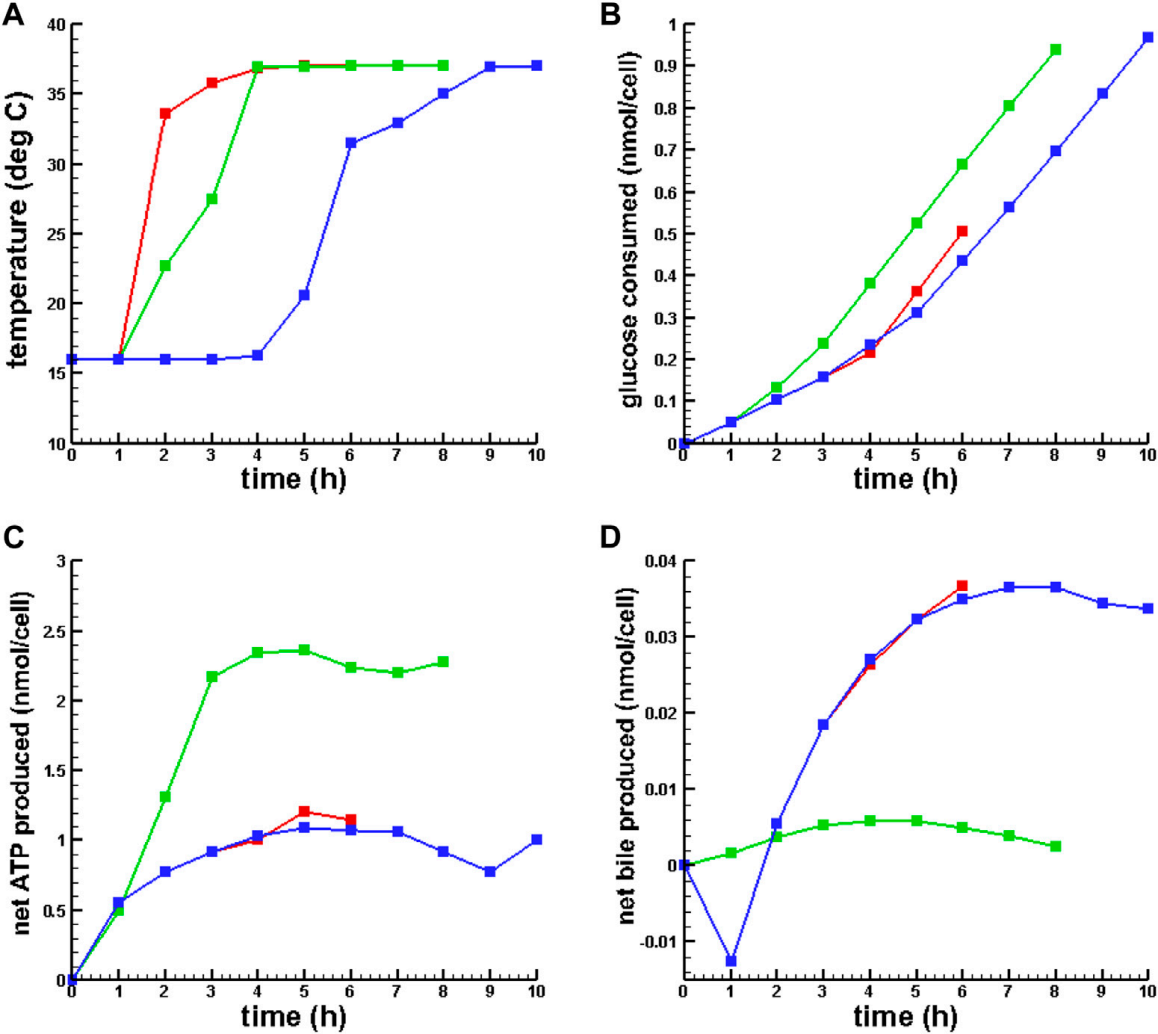
Optimal temperature policies and dynamic behavior of key metabolites for OPT MMP. **(A)** Optimal temperature policy. **(B)** Glucose consumed. **(C)** Net ATP synthesized. **(D)** Net bile produced. 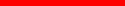
6 h MMP, 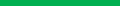
8 h MMP, 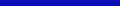
10 h MMP.

Figure 2 presents plots of the optimal temperature policies and the dynamics of a few key metabolites for 6, 8, and 10 h of gradual warming MMP.

Table 3; Figure 2 show that SCS ATP depletion, pH, and energy charge were qualitatively consistent with prior results reported in Lucia et al. (2022) and Bruinsma et al. (2013). While conventional MMP simulation and all MMP with temperature policy optimizations resulted in increased ATP synthesis and energy charge from the same SCS metabolic state, 8 h of gradual warming MMP resulted in significantly more ATP production. Also, concentrations of $H_{2}O_{2}$ following MMP and all OPT MMP were ∼ $1.5x10^{-8}$ M and were approaching the range where, according to Sies (2017), Valdez et al. (2018), Lehtinen and Bonni (2006), Jurczuk et al. (2006), and Thomas et al. (2009), there is potential for oxidative stress. In addition, glutathione depletion was greater in all OPT MMP when compared to conventional MMP and despite the addition of GSH in the SCS UW flush, both MMP and OPT MMP had low glutathione concentration, which is normally in the range 10^−3^ M in a healthy functioning liver.

Also, note that the simulations showed that the net bile synthesis after 8 h of MMP was negative, while for OPT MMP it was positive. Finally, despite the difference in ATP production between MMP and OPT MMP, there was only a slight difference in energy charge.

### 4.3 Optimization of GSH supplementation using hydrogen peroxide perturbation

Note that the UW solution and Williams medium E shown in Table 3 used in the baseline MMP and OPT MMP simulations resulted in hydrogen peroxide concentrations outside the normal range.

Using 6 h of SCS followed by 8 h of OPT MMP with the temperature policy determined in the previous section, an optimization problem was formulated to find the minimum amount of glutathione supplementation needed to keep hydrogen peroxide in the normal range and was defined by Eqs 11, 12:

$$\max_{GSH}\frac{1}{GSH}$$ (11)

such that

$$10^{-9}\;M\leq \left[H_{2}O_{2}\right]\leq 10^{-8}\;M\text{ and }L\leq GSH\leq U\text{ nmol}/\text{cell}$$ (12)

where L and U are lower and upper bounds on the amount of GSH.

In the numerical experiments that follow, the lower bound on GSH was set to $2.22\times 10^{-5}$ nmol/cell and the upper bound was $25.2\times 10^{-5}$ nmol/cell. The initial amount of hydrogen peroxide was perturbed at the start of OPT MMP over the range [1, $300\times 10^{-7}$ nmol/cell], which resulted in initial intracellular hydrogen peroxide concentrations ranging from $0.99\times 10^{-8}$ to $19.15\times 10^{-8}$ M. Oxidative stress and inflammation were measured using the ratio of GSH/GSSG, which is known to decrease as stress and inflammation increase because GHS is consumed and GSSG is synthesized.

#### 4.3.1 Minimum GSH supplementation

**FIGURE 3:**
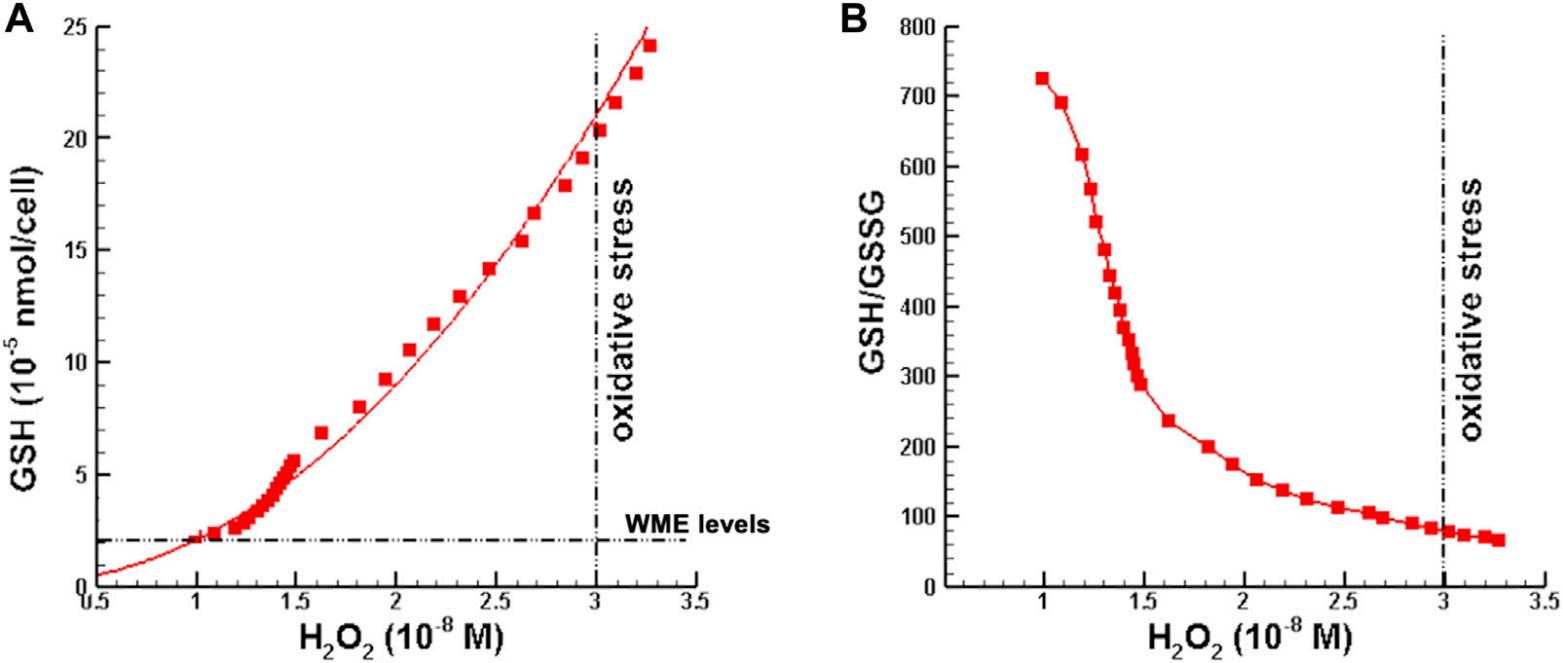
Minimum glutathione requirements as a function of hydrogen peroxide concentration. **(A)** Minimum GSH supplementation such that [H_2_O_2_] is in the normal range at the end of OPT MMP. **(B)** GSH/GSSG ratio corresponding to minimum GSH supplementation. Note that [H_2_O_2_] > $3\times 10^{-8}$ M is defined as oxidative stress (Sies 2017).

In the first set of numerical perturbation studies, initial hydrogen peroxide perturbations were restricted such that H_2_O_2_ concentrations lied in the range from normal to just below the threshold for oxidative stress given in Sies (2017), which is $3\times 10^{-8}$ M. For each perturbation, the minimum amount of GSH required for hydrogen peroxide concentration to be in the normal range at the end of OPT MMP was computed using a Monte Carlo optimization method. Figure 3 is a plot of the minimum GSH supplementation and corresponding GSH/GSSG ratio as a function of hydrogen peroxide concentration. Note that as hydrogen peroxide concentration increases, the minimum amount of GSH needed to keep [H_2_O_2_] in the normal range increases, as one would expect. However, the horizontal line in Figure 3A shows that the amount of glutathione in standard Williams Media E is insufficient when hydrogen peroxide concentration is above the upper limit for the normal range. In Figure 3B the ratio of GSH/GSSG decreases with increasing [H_2_O_2_], indicating movement toward oxidative stress.

**FIGURE 4:**
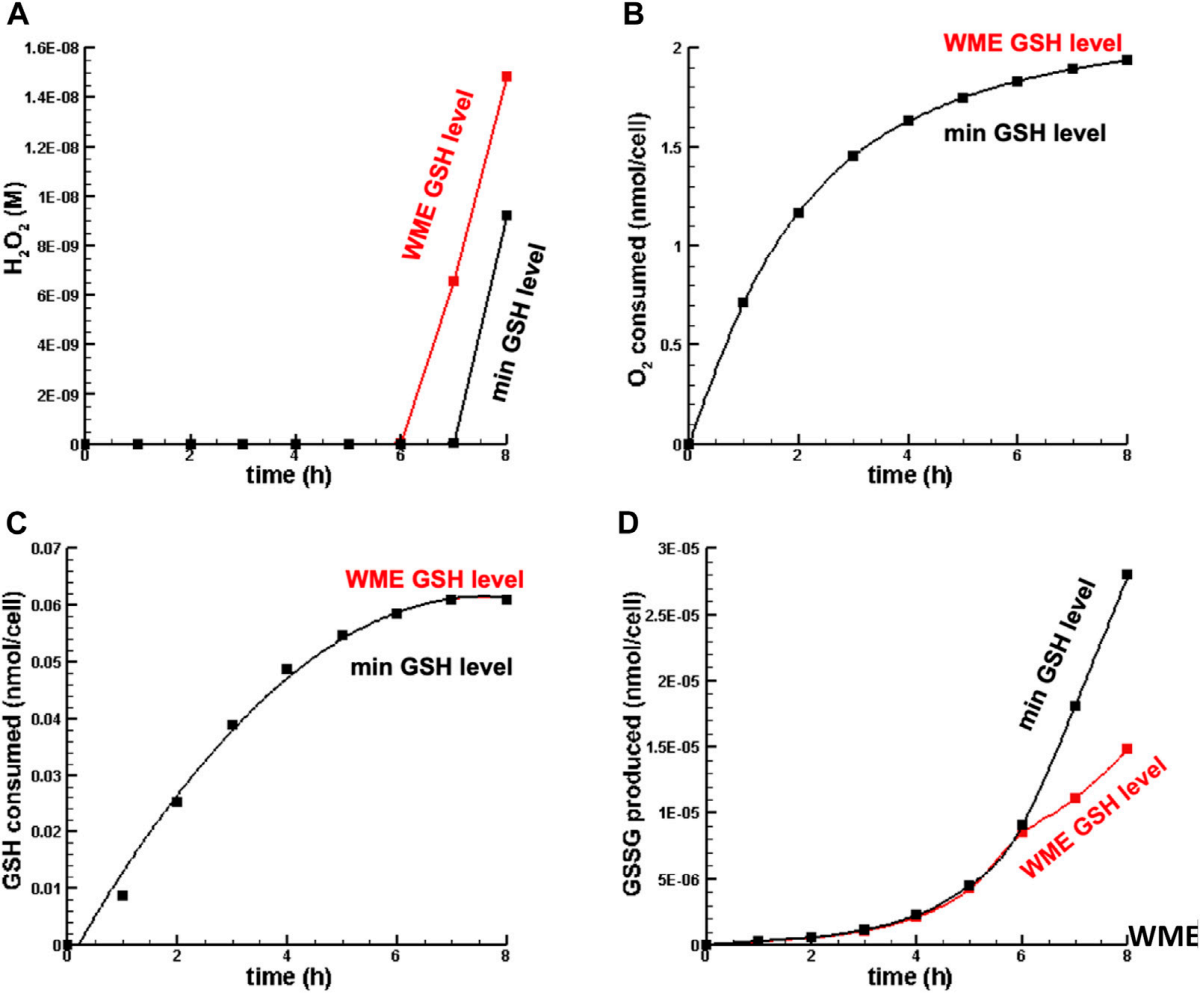
Dynamics of key metabolites in liver cell in oxidative stress. **(A)** H_2_O_2_ concentration. **(B)** O_2_ consumed. **(C)** GSH consumed. **(D)** GSSG synthesized. Note that inf in this figure **(B,C)**, the min GSH level and WME GSH level curves overlap nearly perfectly.

Figure 4 is a plot of the dynamics of [ $H_{2}O_{2}$ ], $O_{2}$ and GSH consumed, and GSSG synthesized for a hydrogen peroxide perturbation of $2.5\times 10^{-7}$ nmol/cell for OPT MMP with WME GSH and with minimum GSH. Note that there is no difference in oxygen and GSH consumed between conventional MMP and OPT MMP, most likely because the levels of hydrogen peroxide were not very different. On the other hand, the most significant difference was in the synthesis of GSSG. OPT MMP produced roughly twice the amount of GSSG, $2.788\times 10^{-5}$ nmol/cell, compared to $1.481\times 10^{-5}$ nmol/cell for conventional MMP.

#### 4.3.2 Inflammation

One fundamental aspect of oxidative phosphorylation is the need for oxygen to produce ATP. Cytochrome C oxidase (or enzyme complex IV) of the electron transport chain (ETC.) is responsible for reducing oxygen (i.e., O_2_ accepts electrons from cytochrome C) and the reduced oxygen is combined with hydrogen ions to form water prior to the production of ATP in complex V (ATP synthase or ATPase). Cyt C oxidase is also responsible for pumping hydrogen ions to the intermembrane space to increase the proton gradient. Protons then re-enter the inner membrane through the F_0_ portion of ATP synthase and are used to power the F_1_ portion (or rotary motor) of ATPase to produce ATP. Unfortunately, oxidative stress/inflammation can lead to cytochrome C oxidase dysfunction (Srinivasan and Avadhami, 2012). This dysfunction can result in reduced useful oxygen consumption (i.e., the amount of oxygen excluding O_2_ converted to superoxide), an increase in water synthesis as enzymes catalase and glutathione peroxidase attempt to remove hydrogen peroxide, a reduction in the proton gradient to drive ATPase, and less ATP.

**FIGURE 5:**
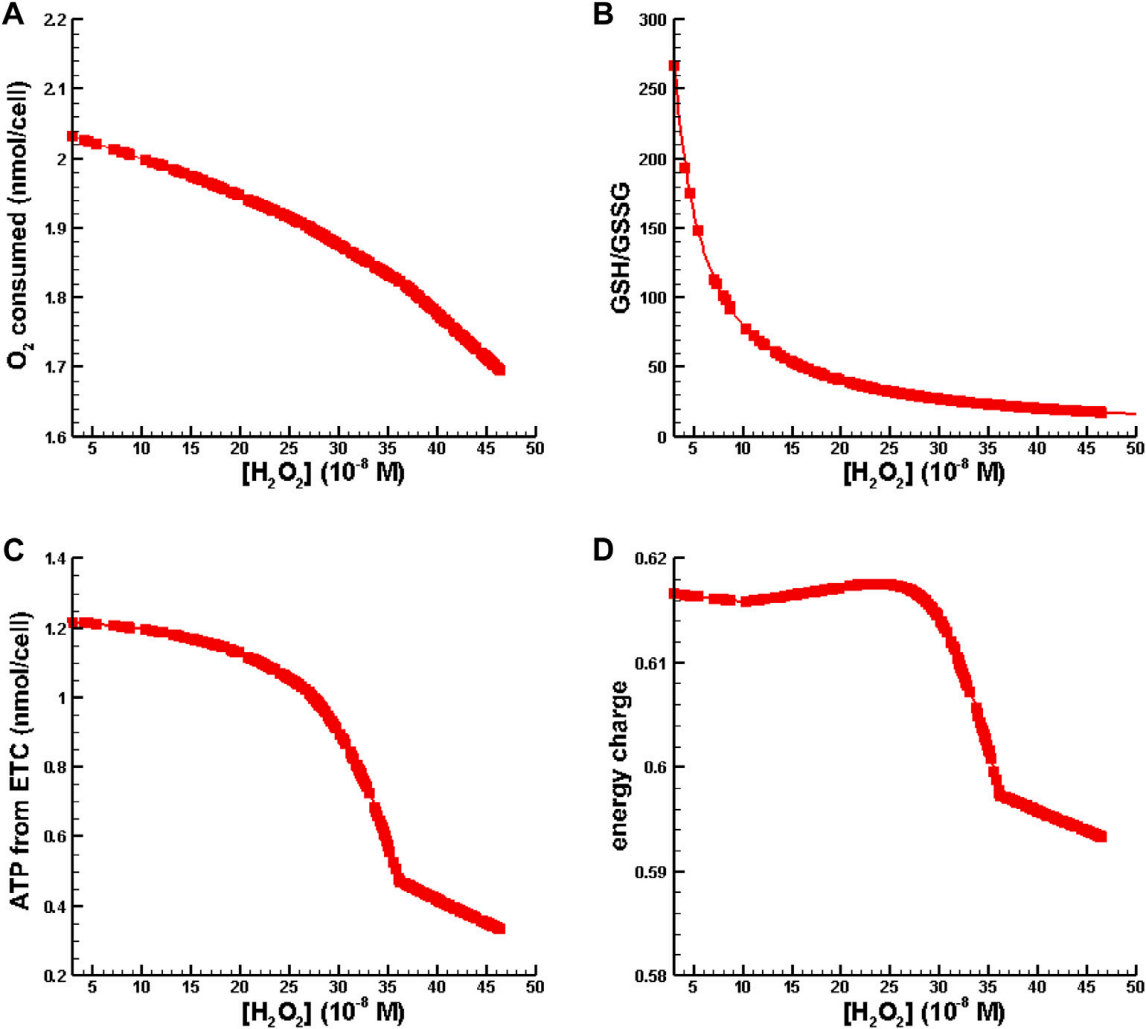
Metabolic behavior in the inflammation regime. **(A)** O_2_ consumed vs. [H_2_O_2_]. **(B)** GSH/GSSG ratio vs. [H_2_O_2_]. **(C)** ATP synthesis from oxidative phosphorylation vs. [H_2_O_2_]. **(D)** Energy charge vs. [H_2_O_2_].

In the second set of numerical experiments, perturbations of hydrogen peroxide were increased to place [H_2_O_2_] in the inflammation regime as defined by Sies (2017) while the total amount of GSH was fixed at the amount available in Williams Medium E (see Table 2). Results for oxygen consumption, ATP synthesis from the, ETC., energy charge, and GSH/GSSG as a function of [H_2_O_2_] are shown in Figure 5.

Figure 5A is indicative of cytochrome c oxidase dysfunction resulting is a reduction of useful oxygen consumption. Figure 5B shows that the conventional biomarker for measuring oxidative stress and inflammation, GSH/GSSG, decreases as a function of [H_2_O_2_]. In Figures 5C, D one can see that ATP synthesis and energy charge decrease with increasing levels of inflammation. All of the behavior shown in Figure 5 is consistent with observations associated with oxidative stress/inflammation.

It is interesting to note that the optimal temperature policy (warming rate) does not change with GSH supplementation. Although the heat of reaction for conversion of hydrogen peroxide to water (Eq. 8) by GSH is exothermic and large ( $∆H_{R}=-196$ kJ/mol), the amount of hydrogen peroxide generated is very small (∼10^−5^ nmol/cell) so the net energy released per cell is ∼2 × 10^−12^ kJ/cell. In addition, small amounts of GSH are consumed and only small amounts of ATP are needed to replenish GSH.

On the other hand, the net amount of ATP generated is on the order of 1 nmol/cell and requires ∼20 kJ/mol. Here the net energy required for ATP synthesis is ∼2 × 10^−8^ kJ/cell, which is four orders of magnitude greater that the energy released by consuming hydrogen peroxide. Therefore, since ATP synthesis has a direct impact on the return function both directly and indirectly through the energy charge (Eq. 8), the dominating energy effects of ATP synthesis compared to the energy generated by hydrogen peroxide consumption show that GSH supplementation has essentially no effect on rewarming rate (or temperature policy).

## 5 Conclusion

In this article the metabolic behavior of a Nash Equilibrium model of liver metabolism under oxidative stress and inflammation during mid-thermic machine perfusion was studied using hydrogen peroxide perturbations. Monte Carlo optimization was used to determine an optimal gradual warming MMP temperature policy (OPT MMP). Using this MMP optimal temperature policy, perturbations in hydrogen peroxide were used to invoke oxidative stress and inflammation. Monte Carlo optimization was used again to determine the minimum amount of glutathione supplementation that kept [H_2_O_2_] in the normal range of $10^{-9}$ to $10^{-8}$ M during oxidative stress. Results also showed that the ratio GSH/GSSG decreased as oxidative stress increased. Additional hydrogen peroxide perturbations were conducted in the inflammation regime. Here oxygen consumed, GSH/GSSG, ATP synthesis from the electron transport chain, and energy charge were computed. Results showed that the metabolic behavior of the liver model in an inflammation state exhibited cytochrome c oxidase (or mitochondrial) dysfunction resulting in reductions in useful oxygen consumption, ATP synthesis, and energy charge per cell.

The medium optimization described in this paper considered the optimization of one compound in Williams Medium E. However, the Monte Carlo procedure described in this work can be extended to find optimal amounts of 1) multiple compounds in Williams Medium E, 2) compounds in other nutrient media such as Histidine-Tryptophan-Ketoglutarate (HTK) or Exsanguinous Metabolic Support (EMS) solution, and 3) therapeutic drugs.

## Figures and Tables

**Table:** 

**ATP**	adenosine triphosphate
**EC**	energy charge
**EMS**	exsanguinous metabolic support
**ETC**	electron transport chain
**G**	Gibbs free energy
**Glu**	glucose
**GSH**	glutathione
**GSSG**	glutathione disulfide
**HTK**	histidine-tryptophan-ketoglutarate
**L**	lower bound
**Mev**	mevalonate
**MP**	machine perfusion
**MMP**	mid-thermic machine perfusion
**NE**	Nash Equilibrium
**OPT MMP**	optimal mid-thermic machine perfusion
**R**	reward function
**SCS**	static cold storage
**T**	temperature
**U**	upper bound
**UW**	University of Wisconsin
**w**	weight
**WME**	Williams Medium E
**[ ]**	concentration

## Data Availability

The raw data supporting the conclusion of this article will be made available by the authors, without undue reservation.

## Supplementary material

SUPPLEMENTARY TABLE S1 Key model assumptions and simplifications.

## References

[B1] Bardallo R. G. Panisello-Rosello A. Sanchez-Nuno S. Alva N. Rosello-Catafau J. Carbonell T. (2022). Nrf2 and oxidative stress in liver ischemia/reperfusion injury. FEBS J. 289, 5463–5479. 10.1111/febs.16336 34967991

[B2] Bruinsma B. G. Berendsen T. A. Izamis M. L. Yarmush M. L. Uygun K. (2013). Determination and extension of the limits to static cold storage using subnormothermic machine perfusion. Int. J Artif Organs 36, 775–780. 10.5301/ijao.5000250 24338652 PMC4091033

[B3] Galaris D. Barbouti A. Korantzopoulos P. (2006). Oxidative stress in hepatic ischemia-reperfusion injury: the role of antioxidants and iron chelating compounds. Curr. Pharm. Des. 12, 2875–2890. 10.2174/138161206777947614 16918418

[B4] Hines I. N. Grisham M. B. (2011). Divergent roles of superoxide and nitric oxide in liver ischemia and reperfusion injury. J. Clin. Biochem. Nutr. 48 (1), 50–56. 10.3164/jcbn.11-016FR 21297912 PMC3022064

[B5] Hines I. N. Harada H. Hoffman J. M. Pavlick K. P. Bharwani S. Wolf R. (2013). “Role of Superoxide in post-ischemic liver injury,” in Madame curie bioscience database (Austin (TX): LandesBioscience). Available from: https://www.ncbi.nlm.nih.gov/books/NBK6357/.

[B6] Jurczuk M. Moniuszko-Jakoniuk J. Rogalaski J. (2006). Evaluation of oxidative stress in hepatic mitochondria of rats exposed to cadmium and ethanol. Pol. J. Environ. Stud. 15 (6), 853–860.

[B7] Laing R. W. Mergental H. Yap C. Kirkham A. Whilku M. Barton D. (2017). Viability testing and transplantation of marginal livers (VITTAL) using normothermic machine perfusion: study protocol for an open-label, non-randomised, prospective, single-arm trial. BMJ Open 7, e017733. 10.1136/bmjopen-2017-017733 PMC5719273 29183928

[B8] Lehtinen M. K. Bonni A. (2006). Modeling oxidative stress in the central nervous system. Curr. Mol. Med. 6, 871–881. 10.2174/156652406779010786 17168738

[B9] Lucia A. DiMaggio P. A. (2019). “Multi-scale computational approach to understanding cancer,” in Data science for healthcare: methodologies and algorithms. Editors Consoli S. Recupero D. R. Petkovic M. (Switzerland AG: Springer Nature), 327–345.

[B10] Lucia A. Ferrarese E. Uygun K. (2022). Modeling energy depletion in rat livers using Nash equilibrium metabolic pathway analysis. Sci. Rep. 12, 3496. 10.1038/s41598-022-06966-2 35241684 PMC8894355

[B11] Lucia A. Uygun K. (2022). Optimal temperature protocols for liver machine perfusion using a Monte Carlo method. IFAC Pap. 55-23, 35–40. 10.1016/j.ifacol.2023.01.011 PMC10895677 38410830

[B12] Miriyala S. Holley A. K. St Clair D. K. (2011). Mitochondrial superoxide dismutase--signals of distinction. Anticancer Agents Med. Chem. 11 (2), 181–190. 10.2174/187152011795255920 21355846 PMC3427752

[B13] Salaris S. C. Barbs C. F. Voorhees W. D. III (1991). Methylene blue as an inhibitor of superoxide generation by xanthine oxidase: a potential new drug for the attenuation of ischemia/reperfusion injury. Biochem. Pharmacol. 42 (3), 499–506. 10.1016/0006-2952(91)90311-r 1650213

[B14] Shibuya H. Ohkohchi N. Seya K. Satomi S. (1997). Kupffer cells generate superoxide anions and modulate reperfusion injury in rat livers after cold preservation. Hepatology 25 (2), 356–360. 10.1053/jhep.1997.v25.pm0009021947 9021947

[B15] Sies H. (2017). Hydrogen peroxide as a central redox signaling molecule in physiological oxidative stress: oxidative eustress. Redox Biol. 11, 613–619. 10.1016/j.redox.2016.12.035 28110218 PMC5256672

[B16] Southard J. H. van Gulik T. M. Ametani M. S. Vreugdenhil P. K. Lindell S. L. Pienaar B. L. (1990). Important components of the UW solution. Transplantation 49 (2), 251–257. 10.1097/00007890-199002000-00004 1689516

[B17] Srinivasan S. Avadhami N. G. (2012). Cytochrome c oxidase dysfunction in oxidative stress. Free Radic. Biol. Med. 53 (6), 1252–1263. 10.1016/j.freeradbiomed.2012.07.021 22841758 PMC3436951

[B18] Tang S.-P. Mao X.-L. Chen Y. H. Yan L. L. Ye L. P. Li S. W. (2022). Reactive oxygen species induce fatty liver and ischemia-reperfusion injury by promoting inflammation and cell death. Front. Immunol. 13, 870239. 10.3389/fimmu.2022.870239 35572532 PMC9098816

[B19] Thomas C. Mackey M. M. Diaz A. A. Cox D. P. (2009). Hydroxyl radical is produced via the Fenton reaction in submitochondrial particles under oxidative stress: implications for diseases associated with iron accumulation. Redox Rep. 14 (3), 102–108. 10.1179/135100009X392566 19490751

[B20] Valdez L. B. Bombicino S. S. Iglesias D. E. Rukavina I. Mikusic A. Boveris A. (2018). Mitochondrial peroxynitrite generation is mainly driven by superoxide steady-state concentration rather than by nitric oxide steady-state concentration. Int. J. Mol. Biol. 3 (2), 56–61. 10.15406/ijmboa.2018.03.00051

