# Supplementary material for: Metabolic perturbation studies using a Nash Equilibrium model of liver machine perfusion: modeling oxidative stress and effect of glutathione supplementation
Source: Front Syst Biol. 2024 Jan 8;3:1260315. doi: 10.3389/fsysb.2023.1260315 (PMC12342001; doi:10.3389/fsysb.2023.1260315)
Supplement: Supplementary file 1 [file Table1.docx]

**Supplementary Table S1: Key Model Assumptions & Simplifications**

| 1 | During cold storage the organ is in a hypoxic state and there is no oxidative respiration. |
| --- | --- |
| 2 | During machine perfusion, the organ is receiving oxygen, so it is not in a hypoxic state. This simplification assumes the oxygen is provided to the periphery as quickly as the central regions. |
| 3 | Under aerobic conditions, since chemical equilibrium represents the upper bound on any overall conversion of glucose to ATP, simple stoichiometry applied to the overall reaction for the Krebs cycle shows that one gets 2 moles of ATP per mole of glucose. |
| 4 | We assume that there is a 2% leakage of O_2_ from the mitochondria to form superoxide. |
| 5 | Consumption of hydrogen peroxide by glutathione must produce glutathione disulfide (Eq. 3), |
| 6 | Inflammation is used strictly as defined by Sies (2017), with intracellular H_2_O_2_ concentration exceeding 0.1μM. Please note that the inflammation involves activation of various transcription factors, cytokine signaling and recruitment of various immune cells, which is not incorporated in our model. |
| 7 | Osmotic equilibrium is assumed in the model, however chemical equilibrium between intra- and extracellular metabolites is not invoked. |
| 8 | Finally, the objective function used for the constrained temperature policy optimization is based on the viability criteria used by Liang et al., 2017 in a clinical study. |
